# Impairment of Proteasome and Autophagy Underlying the Pathogenesis of Leukodystrophy

**DOI:** 10.3390/cells9051124

**Published:** 2020-05-01

**Authors:** Dar-Shong Lin, Che-Sheng Ho, Yu-Wen Huang, Tsu-Yen Wu, Tsung-Han Lee, Zo-Darr Huang, Tuan-Jen Wang, Shun-Jie Yang, Ming-Fu Chiang

**Affiliations:** 1 Department of Pediatrics, Mackay Memorial Hospital, Taipei 10449, Taiwan; 2 Department of Medicine and Institute of Biomedical Sciences, Mackay Medical College, New Taipei 25245, Taiwan; 3 Department of Pediatric Neurology, Mackay Memorial Hospital, Taipei 10449, Taiwan; pedcsho@mmh.org.tw; 4 Department of Medical Research, Mackay Memorial Hospital, Taipei 10449, Taiwan; wendyhuang1219@gmail.com (Y.-W.H.); linws@mmh.org.tw (T.-Y.W.); randy.b746mmh@gmail.com (T.-H.L.); darr.9475@mmh.org.tw (Z.-D.H.); jes53832.f631@mmh.org.tw (S.-J.Y.); 5 Department of Laboratory Medicine, Mackay Memorial Hospital, Taipei 10449, Taiwan; dj.wang@mmh.org.tw; 6 Department of Neurosurgery, Mackay Memorial Hospital, Taipei 10449, Taiwan; 7 Mackay Medicine, Nursing and Management College, Taipei 11260, Taiwan; 8 Graduate Institute of Injury Prevention and Control, Taipei Medical University, Taipei 11031, Taiwan

**Keywords:** leukodystrophies, globoid cell leukodystrophy, psychosine, autophagy, p62, ubiquitin, proteasome

## Abstract

Impairment of the ubiquitin-proteasome-system (UPS) and autophagy causing cytoplasmic aggregation of ubiquitin andp62 have been implicated in the pathogenesis of most neurodegenerative disorders, yet, they have not been fully elucidated in leukodystrophies. The relationship among impairment of UPS, autophagy, and globoid cell leukodystrophy (GLD), one of the most common demyelinating leukodystrophies, is clarified in this study. We examined the ubiquitin and autophagy markers in the brains of twitcher mice, a murine model of infantile GLD, and in human oligodendrocytes incubated with psychosine. Immunohistochemical examinations showed spatiotemporal accumulation of ubiquitin- and p62-aggregates mainly in the white matter of brain and spinal cord at disease progression. Western blot analysis demonstrated a significant accumulation of ubiquitin, p62, and LC3-II in insoluble fraction in parallel with progressive demyelination and neuroinflammation in twitcher brains. In vitro study validated a dose- and time-dependent cytotoxicity of psychosine upon autophagy and UPS machinery. Inhibition of autophagy and UPS exacerbated the accumulation of insoluble ubiquitin, p62, and LC3-II proteins mediated by psychosine cytotoxicity as well as increased cytoplasmic deposition of ubiquitin- and p62-aggregates, and accumulation of autophagosomes and autolysosomes. Further, the subsequent accumulation of reactive oxygen species and reduction of mitochondrial respiration led to cell death. Our studies validate the impairment of proteasome and autophagy underlying the pathogenesis of GLD. These findings provide a novel insight into pathogenesis of GLD and suggest a specific pathomechanism as an ideal target for therapeutic approaches.

## 1. Introduction

Impairment of the ubiquitin proteasome system (UPS) and autophagy have been implicated in the pathogenesis of several neurodegenerative diseases, yet, their role in the pathomechanism of leukodystrophies has not been fully elucidated. Leukodystrophies is a group of devastating neurodegenerative diseases resulting from inherited defects in myelin sheath formation and/or maintenance within the nervous system. The X-linked adrenoleukodystrophy, metachromatic leukodystrophy, and globoid cell leukodystrophy (GLD) caused by lysosomal or peroxisomal enzymes deficiency are the most common demyelinating leukodystrophies; complete rescue therapy is still a challenge and the pathomechanism remains elusive [1,2,3]. Among the leukodystrophies, GLD is the most distinctive type in that greater than 90% of affected individuals are infants [4,5]. Affected infants manifest with developmental delay, hypotonia, and spasticity as early as three months of age, progression involves atrophy, ataxia, epilepsy, cognitive deficits, and eventually death before 2 years of age [4,5]. Currently, GLD is included in newborn screening programs in New York and Kentucky, USA, for rapid diagnosis and early initiation of human stem cell transplantation, though an optimal therapy is still needed [6]. Mechanistic insights into the pathogenesis may prompt a novel therapeutic approach for this disease and other leukodystrophies.

GLD is a lysosomal storage disease caused by an autosomal recessive mutation in the galactosylcerebrosidase (GALC) gene and is characterized by progressive demyelination and astrogliosis in the nervous system [2,4]. It has been shown that enzymatic deficiency of GALC impairs the efficiency of catabolism of galactosylceramide and galactosylsphingosine (psychosine), resulting in progressive accumulation of psychosine in oligodendrocytes, Schwann cells, and neurons. Of note, galactosylceramide does not accumulate in the central nervous system of GLD patients as it can also be degraded by G_M1_ ganglioside β-galactosidase [7], while psychosine can only be hydrolyzed by GLAC and thus accumulates excessively in brain of GLD individuals [4]. The psychosine hypothesis as introduced to explain the pathogenetic mechanism of GLD and has been validated in both cellular and animal models of GLD [8,9]. Cytotoxicity of psychosine causes broad demyelination, reduced remyelination, as well as degeneration of axons and Purkinje cells in the nervous system [4,10,11,12]. To date, many of the studies on pathogenesis and development of therapeutic strategies in GLD have been performed on the naturally occurring twitcher mouse, which is an authentic murine model for infantile GLD [13,14]. Twitcher mice appear normal at birth but start twitching at postnatal day 21, have tremors and steady weight loss around day 25, show apparent limb weakness with kyphotic posture at day 30, have severe hind limb paralysis and intension tremors at day 35, show rapid deterioration after day 40, and early death is usually around day 42 [14,15]. Correlated with the clinical phenotype of twitcher mice, infiltration of macrophages and astrogliosis are first recognized in the cerebellum arbor vita, brain stem, and spinal cord after postnatal day 20, progress to cerebral white matter after day 25, and are evident in cerebral and cerebellar gray matter after day 30. Consistently, demyelination is recognized in cerebellar arbor vita, brain stem, and spinal cord after postnatal day 20, progresses in a caudal-to-rostral order, and is profound in cerebral and cerebellar white matter, brain stem, and spinal cord after day 30 [14,16]. Several therapeutic strategies, including substrate reduction, bone marrow transplantation, gene therapy, and a combination of both cell and transgene delivery, have been applied in twitcher mice and showed variable efficacy, while the complete rescue of phenotype and pathogenesis in GLD is still a challenge [11,17,18,19,20].

Accumulation of toxic metabolite psychosine contributes to the pathogenesis of GLD, though the molecular mechanism is still far from clear. Based on the psychosine hypothesis, several studies have been completed in cells supplemented with exogenous psychosine to unveil the pathological role of psychosine in oligodendrocytes [9,21,22,23,24,25,26,27,28,29,30,31]. It has been shown that psychosine accumulation in membrane microdomains results in the disruption of lipid rafts, alteration of membrane integrity, and inhibition of protein kinase C to the plasma membrane in neurons and oligodendrocytes [22,26]. The preferential accumulation of psychosine in cell membranes causes lipid raft clustering, impairment of tyrosine kinase receptor A-mediated signaling cascades and endocytosis, decreased microtubule stability, and defective axonal transport in neural cells [21,31]. Recently, it has been identified that psychosine accelerates fibrillization of α-synuclein and alters α-synuclein conformation [23]. Moreover, brain samples from twitcher mice and patients affected with the infantile and late-onset forms of GLD have demonstrated neuronal inclusions of α-synuclein, which distributes abundantly in brain areas with the highest levels of psychosine accumulation [23]. The psychosine-mediated alteration of protein conformation and aggregation of misfolded proteins suggest impairment of degradation on misfolded and damaged proteins contributing to the pathogenesis of GLD.

Synthesis of new proteins and degradation of damaged proteins are coordinately regulated and balanced to maintain the cellular protein homeostasis (proteostasis). UPS and macroautophagy (hereafter autophagy) are the two major pathways for eukaryotic intracellular protein catabolism. The UPS is the major protein degradation system and consists of concerted actions of enzymes and ubiquitin to tag damaged proteins for degradation. Enzymatic components E1 and ubiquitin-conjugating E2 enzymes prepare ubiquitin for conjugation, and E3 ligases link the activated ubiquitin from E2s onto the aberrant and/or misfolded soluble proteins, leading to an increasing polyubiquitination chain [32]. The polyubiquitinated proteins are recognized by the 26S proteasome for degradation into small peptides and amino acids. The UPS predominantly degrades short-lived cytosolic and nuclear proteins, including regulator and misfolded proteins as well as damaged proteins. While large misfolded proteins and damaged organelles, which cannot pass through the narrow chamber of the proteasome for degradation, are delivered to and degraded in lysosomes via the process of the autophagy pathway. When autophagy is induced, an isolation membrane emerging from the cellular organelle membrane network engulfs the cargo to form a double-membrane autophagosome, which fuses with the lysosome to form an autolysosome for subsequent lysosomal degradation. The autophagy process is coordinately controlled by autophagy-related (ATG) proteins, among which microtubule-associated protein 1 light chain 3 (LC3), a mammalian Atg8 homolog, undergoes a unique ubiquitin-like conjugation to phosphatidylethanolamine on the autophagic membrane to formLC3-phosphatidylethanolamine conjugate (LC3-II), mediating subsequent autophagosome formation [33]. The autophagy adaptorp62 interacts directly with LC3 to control traffic of ubiquitinated cargo into autophagosomes [34]. During autophagic flux, both LC3 and p62 proteins as well as ubiquitinated cargoes are subject to degradation in autolysosomes. The autophagy essentially degrades long-lived proteins, large protein complexes, aggregated proteins, and organelles. 

Dysfunction of UPS or/and autophagy cause aberrant accumulation of misfolded proteins, which have been linked to pathogenesis in several neurodegenerative diseases, such as Lewy bodies and Lewy neurites in Parkinson’s disease and dementia with Lewy bodies or oligodendroglial cytoplasmic inclusions in multiple system atrophy [35,36,37]. Additionally, impairment of autophagy has also been observed in several lysosomal storage diseases (LSDs), where various stages of the autophagic flux are disrupted in different LSDs [38]. Accumulation of autophagosomes, autolysosomes, and autophagic substrates as well as an impaired lysosomal activity have been demonstrated in a murine model of Niemann–Pick type C1 disease and disease-relevant cells [39], while brain or neurons and astrocytes cultured from Gaucher disease mice have manifested with an accumulation of cytoplasmic aggregates containing ubiquitinated protein, insoluble α-synuclein, and p62 [38]. In GLD, activation of autophagy has been documented in the disease-cell model and cytoplasmic aggregates of p62 in neurons have been observed in the twitcher brain at an early symptomatic stage [24,29,30]. Nonetheless, the impact of psychosine upon autophagy and UPS as well as the role of autophagy and UPS in the pathogenesis of GLD are largely elusive. Herein we show defects in both autophagy and UPS pathways in a murine model of GLD, leading to a progressive accumulation of insoluble ubiquitin and p62 proteins concomitant with cytoplasmic aggregates of p62 and ubiquitin in the brain at disease progression. Furthermore, an in vitro study using human oligodendrocyte MO3.13 cells validates a time- and dose-dependent cytotoxicity of psychosine upon UPS and autophagy, and recapitulates the pathogenesis observed in the GLD murine model. Our work, therefore, suggests that psychosine-mediated impairment of UPS and autophagy underlies the pathogenesis of GLD.

## 2. Materials and Methods

### 2.1. Animals

The heterozygous (*twi*/+) twitcher mice with a congenic C57Bl/6 background were obtained from the Jackson Laboratory (Bar Harbor, ME, USA) and maintained under standard housing conditions in the animal research facility of our institution. Wild-type (+/+) and homozygous (*twi*/*twi*) twitcher mice were generated by strict inbred mating of heterozygous twitcher mice. Genomic DNA was isolated from tissue sample of 2-day-old pups. The genotypes were determined in genomic DNA by a PCR amplification with a sense primer 5′-ATGAGACTGAAATTGGTAGACAGC-3′ and an anti-sense primer 5′-ATGGCCCACTGTCTTCAGGTGATA-3′ specific for the mutant allele, followed by EcoRV digestion as previously described [40,41]. All animal procedures were approved and performed according to the guidelines established by the Animal Care and Use Committee of our institution. Mice were euthanized under anesthesia with pentobarbital. Brains and spinal cords were removed immediately, snapped frozen with liquid nitrogen, and sorted at −80 °C before biochemical analysis. Alternatively, the hemisphere and spinal cords were post-fixed in 4% paraformaldehyde (PFA) overnight at 4 °C, cryoprotected in 30% sucrose, and then quickly frozen in OCT compound (TissueTek, Sakura Finetek, Torrance, CA, USA). Cryosectioned slices of 8-µm thickness were obtained and stored at −80 °C before immunohistochemistry.

### 2.2. Cell Culture and Treatment

Human oligodendrocytes MO3.13 cells were maintained in Dulbecco’s modified eagle’s medium (DMEM; Invitrogen, Eugene, OR, USA),supplemented with 10% (*v*/*v*) fetal bovine serum (FBS; Gibco, Rockford, IL, USA) and 1%penicillin G/streptomycin sulfate at 37 °C in a humidified atmosphere of 5% (*v*/*v*) CO_2._When indicated, theMO3.13 cells were plated in standard 6-well cell culture plates overnight. The next day, MO3.13 cells were exposed to different concentrations of psychosine (Sigma-Aldrich, St. Louis, MO, USA)/ DMSO (Sigma-Aldrich) for 24 h. For the selective experiment, MO3.13 cells were treated with or without 20 µM psychosine for 72 h in the presence of inhibitors 20 µM chloroquine (Sigma-Aldrich) and/or 0.25 µM MG132 (Sigma-Aldrich) during the last 24 h.

### 2.3. Immunofluorescence Staining

Brain cryosections were fixed with 4% PFA for 20 min, microwave heated in Tris-ETDA pH9 buffer for 3 min, quenched with 3% H_2_O_2_ in methanol for 5 min, and permeabilized with 0.5% Triton X-100 for 10 min. The sections were then blocked in 5% horse serum/0.25% Triton X-100 in phosphate-buffered saline (PBS) for 30 min at room temperature and incubated with primary antibodies (Table 1) over-night at room temperature. After extensive washing with PBS, the sections were visualized with secondary antibodies conjugated with Alex Flour 488 or Alex Flour 594 (Molecular Probes, Eugene, OR, USA) and counterstained with DAPI.

Cultured cells were fixed with 4% paraformaldehyde in PBS for 10 min at room temperature, washed extensively with PBS, then permeabilized with cold methanol for 10 min, microwave heated in Tris-ETDA pH9 buffer for 3 min, and washed with PBS. Cells were blocked with 10% goat serum/0.25% Triton X-100/PBS for 30 min at room temperature, incubated with primary antibodies (Table 1) for 2h at room temperature, washed 3 times with PBS, then incubated with secondary antibodies conjugated with Alex Flour 488 or Alex Flour 594 (Molecular Probes) for 1 h at room temperature. The cells were washed 3 times with PBS, counterstained with DAPI, and visualized under a fluorescence microscope.

### 2.4. Preparation of Protein Samples

Proteins were extracted from deep-frozen tissues and cells, respectively, lysed with T-PER™ Tissue Protein Extraction Reagent (ThermoFisher Scientific) supplemented with Halt^TM^ protease inhibitor cocktail (Thermo Fisher Scientific). The tissue was then homogenized in Eppendorf Scientific tubes with a pellet pestle at 4 °C. The lysate was centrifuged at 17,000× *g* for 20 min at 4 °C and the supernatant was collected as a detergent soluble fraction according to methods described previously [42]. The insoluble pellet was dissolved in T-PER™ Tissue Protein Extraction Reagent supplemented with 2% sodium dodecyl sulfate (SDS; Merk, Darmstadt, Germany), sonicated on ice, and centrifuged at 17,000× *g* for 20 min at 4 °C. The supernatant was collected as an insoluble fraction [42]. The concentration of each protein fraction was measured using the BCA protein assay (Thermo Fisher Scientific, Rockford, IL, USA) and subjected to Western blotting analysis.

### 2.5. Western Blot Analysis and Immunoprecipitation

Each protein sample was subjected to sodium dodecyl sulfate-polyacrylamide gel electrophoresis (SDS-PAGE) using10% or 12% polyacrylamide gels, and transferred onto a Poly-vinylidenedifluoride (PVDF) membrane (Millipore). The membranes were blocked with 0.5% skimmed milk in TBST (20 mM Tris-HCl, pH 7.5, 150 mM NaCl, 0.1% Tween20) for 1 h at room temperature, washed with TBST, and immunoblotted with the primary antibody (Table 1) in TBST overnight at 4 °C. The PVDF membrane was then washed, incubated with a secondary antibody conjugated to horseradish peroxidase for 2 h at room temperature, washed again, and then visualized by Immobilon Western Chemiluminescent HP Substrate (Millipore), which provides high sensitivity over a broad detection range, and detected by ChemiDoc-It 815 Imaging System (Analytik Jena, Upland, CA, USA), which provides a broad dynamic range of imaging for linear ranges of detection. To avoid oversaturation of signal intensity, multiple images with different exposure times were captured sequentially by ChemiDoc-It 815 Imaging System and the image with the best results was selected for quantitative analysis by VisionWorks software (Analytik Jena). Furthermore, if a protein of interest was expressed at relatively low or high levels, the amount of the loaded sample was increased or diluted, accordingly, to fit within the linear range of detection.

To verify the interaction between insoluble p62 and ubiquitin, immunoprecipitation (IP) was performed using Pierce protein A/G magnetic beads (Thermo Fisher Scientific) and magnetic bead-based separation according to the manufacturer’s protocol. Briefly, the anti-p62/anti-ubiquitin antibody was added to prepared protein A/G magnetic beads and then mixed and incubated on a rotating platform for 15 min at room temperature. The magnetic beads were collected and washed with Modified Coupling Buffer three times, then incubated with disuccinimidyl suberate for 30 min. The antibody-crosslinked magnetic beads were washed three times with Elute Buffer followed by two washes with Modified Coupling Buffer. The lysates were incubated with antibody-crosslinked magnetic beads overnight at 4 °C. The beads were washed twice with Modified Coupling Buffer and once with ultrapure water and then incubated with Elute Buffer for 5 min at room temperature. The eluate containing the target antigen was collected by magnetic separation with beads, and neutralized with neutralization buffer. The IP products were detected by Western blot analysis as described above.

### 2.6. Detection of Autolysosomes

Briefly, MO3.13 cells treated with or without psychosine in the presence of autophagy and/or proteasome inhibitors were stained with Cyto-ID Green detection reagent (Enzo Life Science, Taipei, Taiwan) and LysoTrakcer Red (Thermo Fisher Scientific) and counterstained with Hoechst 33342, according to the manufacturer’s protocol. Images were obtained and recorded using the ImageXpress Micro 4 system (Molecular Devices) at 40× magnification in 18 fields of view per well and analyzed by the Multi-Wavelength Cell Scoring Application Module. The cells were selected based on both Cyto-ID and LysoTracker Red fluorescence by setting the maximal and minimal diameters and the minimal fluorescence intensity relative to the background from both channels. The integrated intensity/cell which represented the fluorescence of each cell was used to measure Cyto-ID and LysoTracker Red co-expression in different groups. Colocalization of both Cyto-ID and LysoTracker Red fluorescence corresponded to the puncta of autolysosomes.

### 2.7. Reactive Oxygen Species (ROS) Detection 

Levels of reactive oxygen species (ROS) were measured using the fluorescent probe MitoSOX™ Red according to the manufacture’s protocol (Thermo Fisher Scientific). Briefly, theMO3.13 cells were cultured in a 6 cm dish and treated without or with psychosine/Chloroquine/MG132 in a humidified atmosphere of 5% (*v*/*v*) CO_2_ at 37 °C. Cells were harvested by trypsinization, incubated with MitoSOX™ Red for 10 min at 37 °C in the dark, and centrifuged. Cells were resuspended in pre-warmed Hank’s Balanced Salt Solution (HBSS), centrifuged, washed with pre-warmed HBSS, centrifuged again, and resuspended in pre-warmed HBSS. Cells were then analyzed on a flow cytometer for ROS detection, using the 510 nm laser for excitation and detected at 580 nm.

### 2.8. Mitochondrial Respiration

Bioenergetic profiles were determined using theXF24 extracellular flux analyzer (Seahorse Biosciences, Lexington, MA, USA). MO3.13 cells were seeded on XF24 cell culture microplates and maintained in a humidified atmosphere of 5% (*v*/*v*) CO_2_ at 37 °C. The MO3.13 cells were treated without or with psychosine/ Chloroquine/MG132 as previously described. Before assay, MO3.13 cells were equilibrated in unbuffered DMEM medium (Gibco) supplemented with 25 mM glucose, 1 mM sodium pyruvate, and 2 mM L-Glutamine and transferred to a non-CO2 incubator for 1 h before measurement. Mitochondrial oxygen consumption rate (OCR) was measured in real-time for 20 min. Each plotted value of real-time assessment of mitochondrial respiration was normalized with cell number and results were presented as mean ± S.D.

### 2.9. Cell Viability Assay

The MO3.13 cells were seeded on a 96-well plate and treated as previously described in a humidified atmosphere of 5% (*v*/*v*) CO_2_ at 37 °C. Cell numbers were quantified by the CyQUANT cell proliferation assay kit according to the manufacture’s protocol (Thermo Fisher Scientific). Briefly, cells were incubated with CyQUANT dye at 37 °C for 60 min and fluorescence intensity was measured on a plate reader at OD = 530nM.

### 2.10. Statistical Analysis

All data were obtained from at least three independent experiments and results were expressed as the mean ± S.D., unless stated otherwise. Student’s *t*-test was used for two-group comparison and ANOVA test was used for multiple comparisons; *p*-values less than 0.05 were considered significant. 

## 3. Results

### 3.1. Cytoplasmic Aggregates Containing Ubiquitin and p62 in the Twitcher Brain

**Figure 1:**
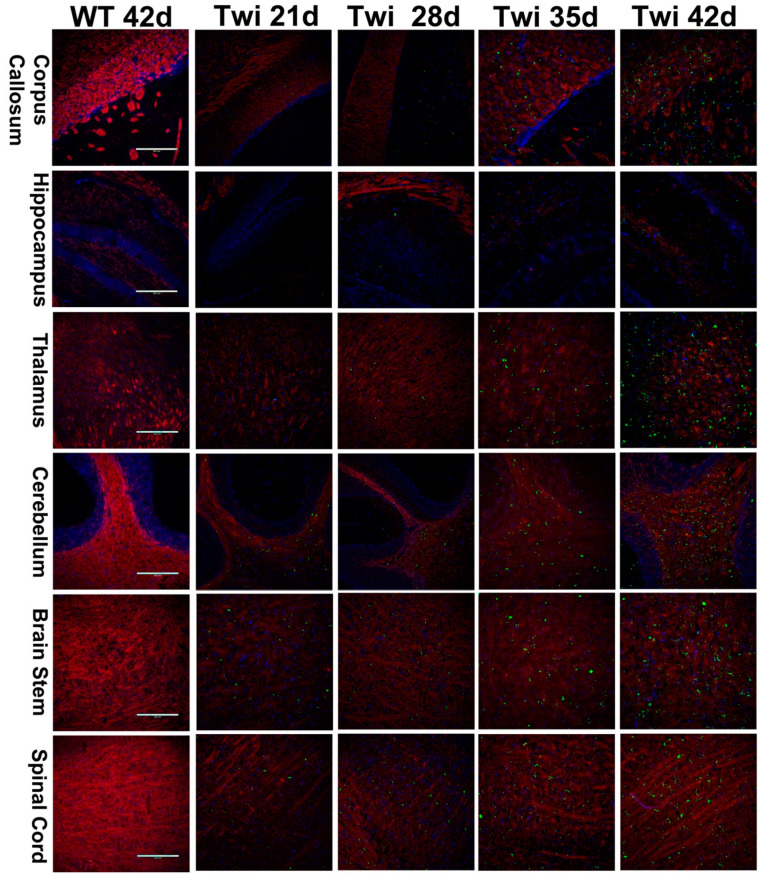
Spatial and temporal distribution of ubiquitin aggregates in the brain of twitcher mice. Distribution of ubiquitin aggregates in different regions of the twitcher (Twi) brain at ages 21 to 42 days. The twitcher brain and wild-type (WT) brain were immuno-stained with anti-ubiquitin (in green) and anti-PLP (in red), respectively, and nuclei were counterstained with DAPI (in blue). Scale bars: 200 μm.

**Figure 2:**
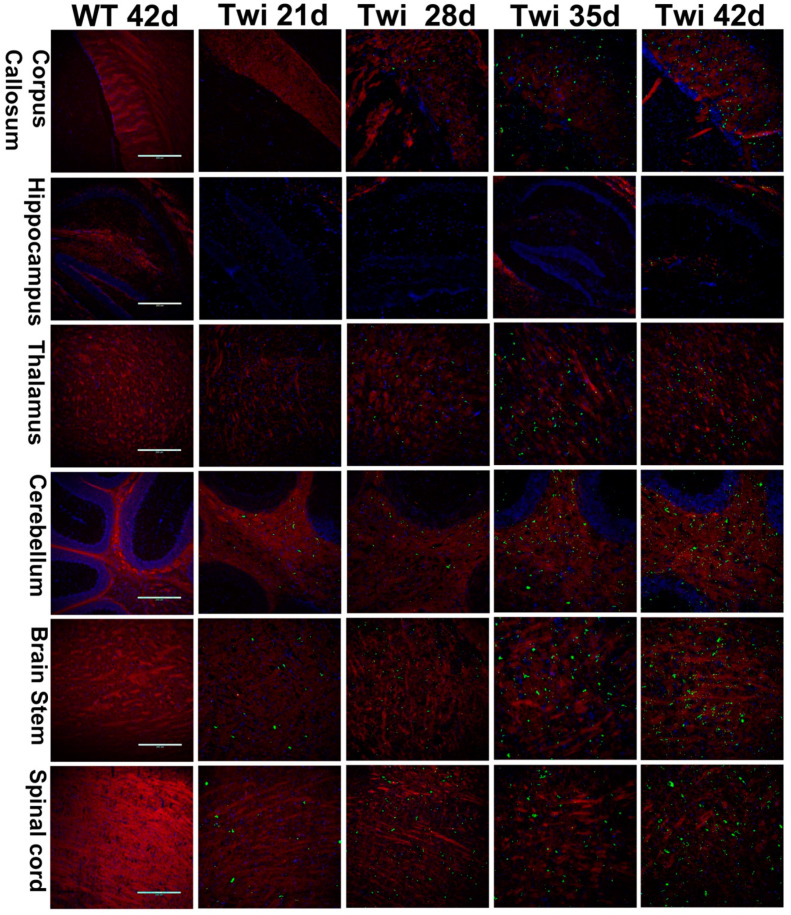
Progressive accumulation of p62 aggregates in the brain of twitcher mice. Distribution of p62 aggregates in different regions of the twitcher (Twi) brain at ages 21 to 42 days. The twitcher brain and wild-type (WT) brain were immuno-stained with anti-p62 (in green) and anti-PLP (in red), respectively, and nuclei were counterstained with DAPI (in blue). Scale bars: 200 μm.

Dysfunction of the UPS and autophagy impairs protein homeostasis leading to accumulation of toxic and misfolded proteins and ubiquitin-positive aggregates in neurons, which is a pathologic hallmark of many neurodegenerative diseases [35,36,37,38]. To investigate whether dysfunction of both UPS and autophagy contribute to the pathogenesis of GLD, we first examined the expression patterns of ubiquitin and p62, molecular indicators of UPS and autophagy, respectively, in the brain of twitcher mice. Antibodies directed against ubiquitin and p62 were used in the analysis of immunohistochemistry. For the first time, spatiotemporal accumulation and distribution of ubiquitin- and p62-aggregates during the progression of the disease were documented in the twitcher brain (Figure 1 and Figure 2). At postnatal day 21, ubiquitin aggregates appeared sparsely in the spinal cord, brain stem, and cerebellum arbor vita, and aggregates were observed scarcely in the corpus callosum (Figure 1). At postnatal day 28, the accumulation of ubiquitin aggregates was more evident in the brain stem, spinal cord, and cerebellum arbor vitae. Ubiquitin aggregates were detected moderately in the corpus callosum, septum, thalamus, and hypothalamus, and scarcely in the cortical layer VI along the corpus callosum and in granular and molecular layers of the cerebellum (Figure 1). At postnatal day 35, variable puncta of ubiquitin aggregates distributed diffusely in the spinal cord, brain stem, cerebellum arbor vitae, and corpus callosum. The thalamus, hypothalamus, and septum demonstrated more evident distribution of ubiquitin aggregates (Figure 1). At postnatal day 42, ubiquitin aggregates were abundant in the spinal cord, brain stem, cerebellum arbor vitae, cerebellar peduncle, fornix, internal capsule, and corpus callosum. The cortex and granular and molecular layers of the cerebellum showed moderate ubiquitin aggregates. Ubiquitin aggregates were observed scarcely in the hippocampus (Figure 1).

Previous studies have observed the presence of p62 aggregates at postnatal day 20 and day 30 in the brain of twitcher mice, and suggested the dysregulation of autophagy as a molecular pathogenesis of GLD [29]. However, the anatomical and temporal distribution of p62 aggregates in the twitcher brain has not been elucidated. Using immunohistochemistry staining of the twitcher brain at different postnatal days, we were able to determine the regional and temporal distribution of p62 aggregates (Figure 2). Intriguingly, the distribution of p62 aggregates was in line with that of ubiquitin aggregates in the twitcher brain. P62 aggregates were detected sparsely in the brain stem, spinal cord, and cerebellum arbor vitae at postnatal day 21, then accumulated progressively and were abundant in these regions as well as in the fornix, internal capsule, and cerebellar peduncle at postnatal day 42. While in regions of the thalamus, hypothalamus, and septum, p62 aggregates were sparse at postnatal day 28 and were more evident at postnatal day 42. Consistent with the aggregation of ubiquitin in twitcher brains, p62 aggregates were rare in the cerebral cortex and granular and molecular layers of cerebellum at postnatal day 28, and were moderate at postnatal day 42. While the hippocampus showed scarce p62 aggregates at postnatal day 42. Overall, the spatiotemporal distribution of p62 aggregates was identical to the progressive aggregation of ubiquitin in twitcher brains. 

### 3.2. p62 Colocalized with Ubiquitin in Aggregates

**Figure 3:**
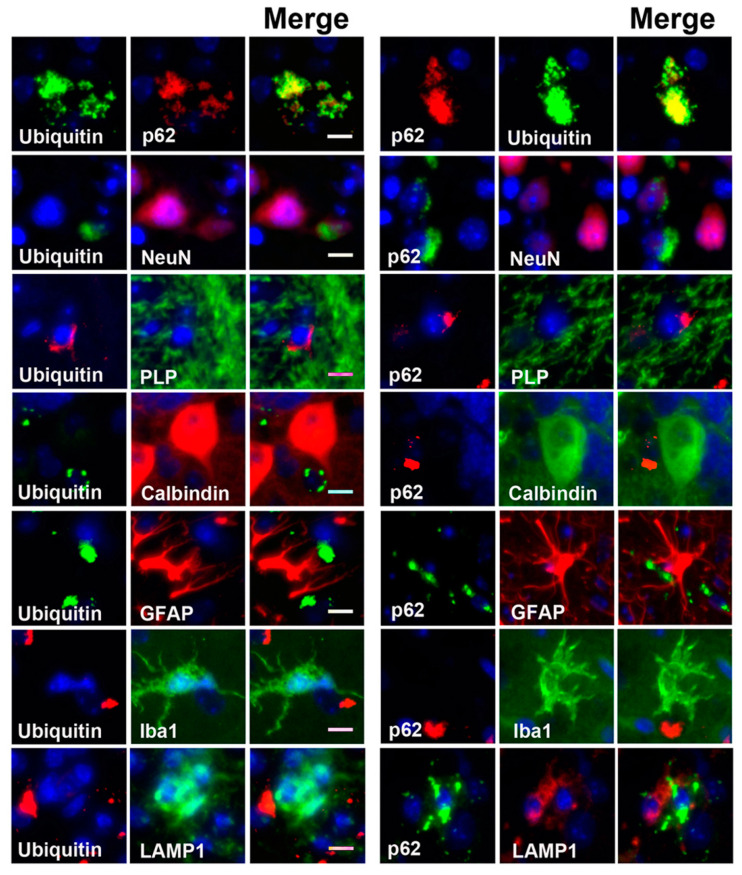
Accumulation of ubiquitin/p62 aggregates in oligodendrocytes and neurons. Sections of twitcher brain were double labeled with anti-ubiquitin/anti-p62 and anti-NeuN (neuron)/anti-PLP (oligodendrocytes)/anti-calbindin (Purkinje cells)/anti-GFAP (astrocyte)/anti-Iba1 (microglia)/anti-LAMP1 (lysosome), respectively, and nuclei were counter-stained with DAPI (blue). Scale bars: 10 μm.

Ubiquitin colocalized with p62 in cytoplasmic inclusions has often been documented in several neurodegenerative disease pathologies [43]. Thus, to further investigate whether the ubiquitin colocalized with p62 in cytoplasmic aggregates as observed in our study, the sections of twitcher brain were double immunolabeled for p62 and ubiquitin with specific antibodies, respectively (Figure 3). Intriguingly, our results revealed that ubiquitin-positive signals were almost exclusively colocalized with p62 in cytoplasmic aggregates at all time points from postnatal day 21 to day 42. Further, p62- and ubiquitin-aggregates accumulated progressively and widely distributed in the brain of twitcher mice in a caudal-to-rostral axis as the disease progressed in line with the deterioration of demyelination and phenotype, suggesting the correlation between disturbed proteostasis and pathogenesis in GLD. However, p62- and ubiquitin-aggregates were not colocalized with lysosome marker LAMP1 (Figure 3), suggesting the deposition of cytoplasmic aggregates as a result of impaired proteostasis.

### 3.3. Colocalization of Aggregates in Degenerative Neurons and White Matter

To investigate the localization of ubiquitin and p62 aggregates, neuronal cells and brain slices from wild-type and twitcher mice were double immunolabeled with anti-ubiquitin or anti-p62 together with antibodies specific for different brain cell types to visualize astrocytes, microglia, oligodendrocytes, neurons, and Purkinje cells (Figure 3). A previous study by Del Grosso and colleagues revealed the colocalization of p62 aggregates in neurons at the early symptom stage (postnatal day 20) of twitcher mice, albeit the extent and affected regions were not indicated [29]. In the present study, we further documented that ubiquitin- and p62-aggregates are seen conjointly with the neurons in the brain stem, spinal cord, and granular layer of cerebellum and were sparsely found at postnatal day 28 and moderately at day 42 (Figure 3). Moreover, neurons with either ubiquitin or p62 aggregates showed attenuated signals of both nuclei and neuronal protein marker NeuN, indicating the progression of neuronal degeneration.

However, ubiquitin- and p62-aggregates were not detected in either astrocytes or microglia (Figure 3). Further, in the present study, Purkinje cells were devoid of cytoplasmic aggregates (Figure 3), and this supports our previous study showing atrophy of Purkinje cells and neural degeneration in the twitcher brain [11]. It has been assumed that the degeneration of granule cells leads to lower secretion of neurotrophic factors, which are required for modulating the dendritic differentiation of Purkinje cells and cerebellar plasticity, and the absence of which would lead to impaired dendritogenesis, synaptogenesis of Purkinje cells, and subsequently cell death [11,44,45].

Intriguingly, ubiquitin- and p62-aggregates were almost exclusively localized in the white matter of the nervous system (Figure 1, Figure 2 and Figure 3), including the corpus callosum, fornix, internal capsule, cerebellum arbor vitae, cerebellar peduncle, fiber plexus, bundles in the brain stem and spinal cord, as well as fiber arrays in the cortex, midbrain, thalamus, and hypothalamus. More specifically, small puncta of aggregates were localized in the perinuclear region of oligodendrocytes (Figure 3). While large pleomorphic aggregates were embedded among corrupted fiber bundles of white matter, they were more discernible as demyelination progressed (Figure 3). The progressive accumulation of ubiquitin- and p62-aggregates in the white matter is paralleled by the progression of demyelination and deterioration of phenotype, implicating cytotoxicity of inclusions upon oligodendrocyte viability contributes to the progressive demyelination in GLD.

### 3.4. Dysfunction of Autophagy and the Proteasome-Ubiquitin System in the Twitcher Brain

**Figure 4:**
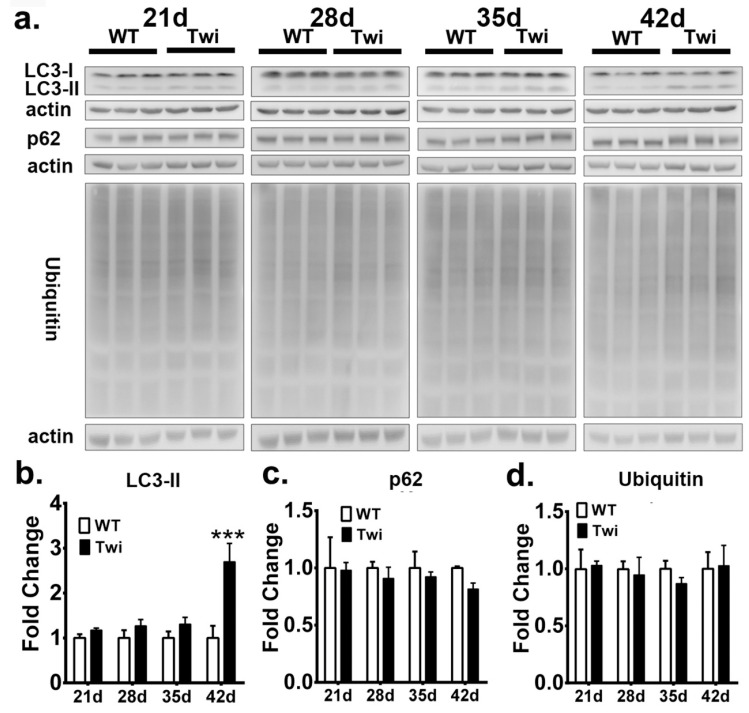
Increased autophagic flux in twitcher brain. **a** Western blot analysis of the total LC3-II, p62, and ubiquitin content in brain stems from wild-type (WT) and twitcher (Twi) mice at ages 21 days, 28 days, 35 days, and 42 days. Soluble fraction was analyzed and β-actin (actin) was used for the loading control. **b**, **c**, **d** Quantification of soluble LC3-II (**b**), p62 (**c**), and ubiquitin (**d**). Values are expressed as mean ± S.D. (*n* = 3). Statistical significance ***p < 0.001.

**Figure 5:**
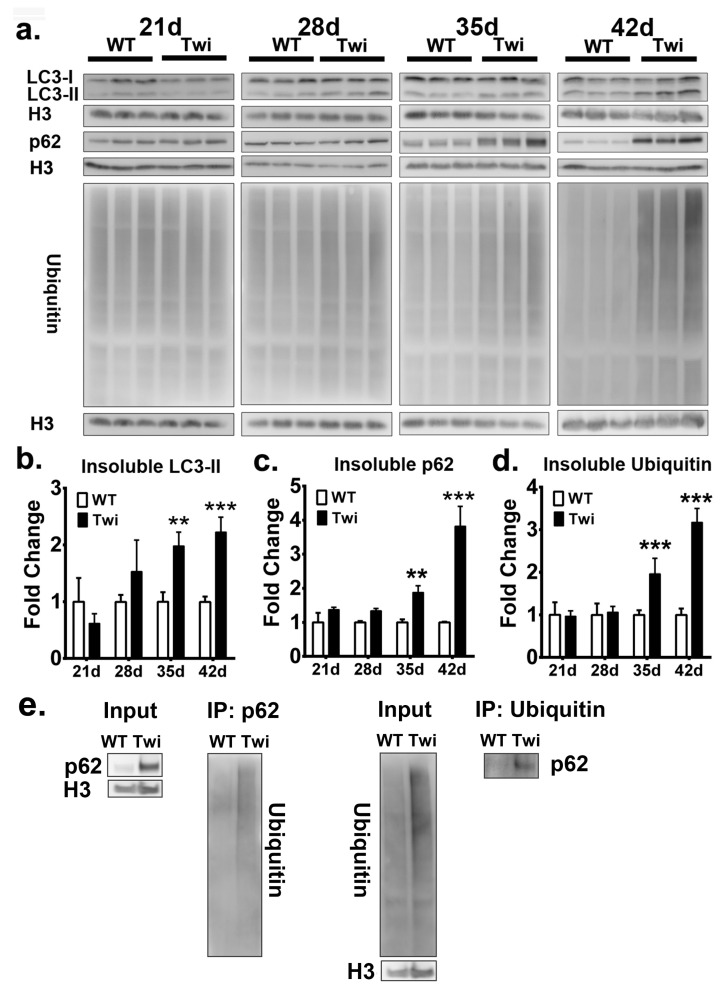
Defective autophagy and ubiquitin-proteasome system in twitcher brains. **a:** Western blot analysis of the total LC3-II, p62, and ubiquitin content in brain stems from wild-type (WT) and twitcher (Twi) mice at ages 21 days, 28 days, 35 days, and 42 days. Insoluble fraction was analyzed and histon-H3 (H3) was used for the loading control. b, c, d Quantification of insoluble LC3-II (**b**), p62, (**c**) and ubiquitin (**d**). Interaction between insoluble p62 and ubiquitin was verified by immunoprecipitation analysis (**e**). Values are expressed as mean ± S.D. (*n* = 3). Statistical significance, **p < 0.01, ***p< 0.001.

To biochemically investigate the molecular markers of autophagy and UPS, we extracted detergent soluble and insoluble proteins from the brain stem of the twitcher mice and wild-type (WT) mice at postnatal days 21, 28, 35, and 42, and quantified the expression of LC3, p62, and ubiquitin after Western blot analysis (Figure 4a and Figure 5a). 

The quantitative analysis of molecular markers of autophagy in fractions of soluble proteins showed that LC3 II was significantly increased in the twitcher brain at postnatal day 42 when compared to that of the WT brain (Figure 4b), while the levels of p62 in the twitcher brain at all time point were compatible with that of the WT brain (Figure 4c), indicating the activation of autophagy. Similarly, the level of ubiquitin between WT and twitcher brains was identical at all time points (Figure 4d). Intriguingly, the quantitative analysis of the insoluble protein fractions revealed a prominent accumulation of LC3 II, p62, and ubiquitin in twitcher brains compared to that of WT brains, suggesting dysfunction of autophagy and UPS (Figure 5b–d). Levels of insoluble LC3 II, p62, and ubiquitin were significantly increased in the twitcher brain at postnatal day 35 and day 42 (Figure 5b–d), when the twitcher mice showed a rapid deterioration of clinical phenotypes. Further, the binding of insoluble p62 and ubiquitin was confirmed by the immunoprecipitation analysis (Figure 5e). Taken together with our immunofluorescent analysis, it is concluded that ubiquitin and p62 inclusions are insoluble, cytosolic protein aggregates. The accumulation of insoluble ubiquitin proteins and cytoplasmic deposition of ubiquitin-positive aggregates suggests accumulation of oligomerized protein aggregates and indicates defects of UPS commonly associated with many neurodegenerative proteinopathies. While the accumulation of LC3 II and p62 in insoluble protein fractions together with cytoplasmic p62 aggregates indicates interruption of autophagic flux, the accumulation of insoluble ubiquitin and broad deposition of ubiquitin aggregates implicates the dysfunction of UPS.

**Figure 6:**
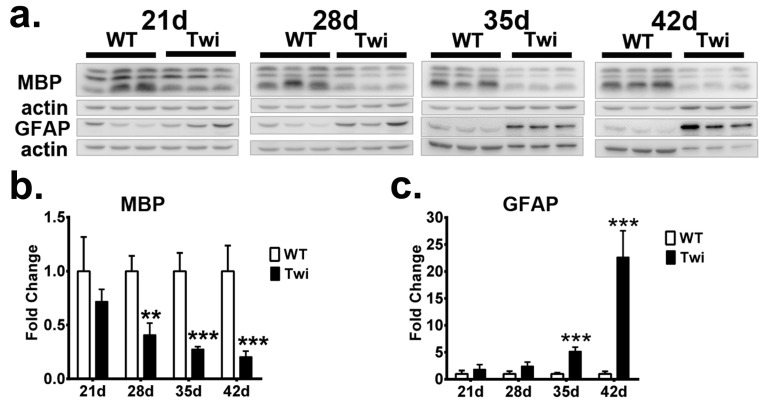
Progressive astrocytosis and demyelination in twitcher brain. (**a**) Western blot analysis of the total MBP and GFAP content in brain stems from wild-type (WT) and twitcher (Twi) mice at ages 21 days, 28 days, 35 days, and 42 days of age. **b**, **c** Quantification of MBP (**b**) and GFAP (**c**). β-actin (actin) was used for the loading control. Values are expressed as mean ± S.D. (*n* = 3). Statistical significance, **p< 0.01, ***p < 0.001.

Additionally, the expressions of GFAP and MBP proteins, molecular markers of astrocytes and myelin, respectively, were also determined in both twitcher and WT mice (Figure 6). In concordance with the progression of neuroinflammation and demyelination in the pathogenesis of GLD, an increasing level of GFAP and decreasing expression of MBP were demonstrated in the twitcher brain when compared to those of a WT brain. Accordingly, accumulation of insoluble p62 and insoluble ubiquitin concomitant with the formation of p62- and ubiquitin-aggregates in the twitcher brain were in line with the progression of neuroinflammation, demyelination, and the deterioration of the disease.

### 3.5. Psychosine-Induced Autophagy and Cell Death in Time-and Dose-Dependent Toxicity

**Figure 7:**
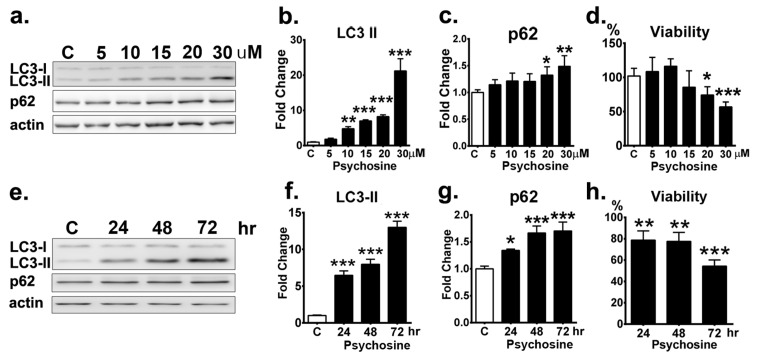
Dose- and time-dependent cytotoxicity of psychosine upon MO3.13 cells. (**a**) Western blot analysis of total LC3-II and p62 in MO313 cells treated with different concentration of psychosine (psy) for 24 h. β-actin (actin) was used for the loading control. (**b**,**c**,**d**) Quantification of total LC3-II (**b**) and p62 (**c**) and cell viability (**d**). e Western blot analysis of total LC3-II and p62 in MO313 cells treated with 20 µM psychosine for 72 h. (**f**,**g**,**h**) Quantification of total LC3-II (f) and p62 (**g**) and cell viability (**h**). Values are expressed as mean ± S.D. (Western blot, *n* = 3; Viability, *n* = 6). Statistical significance *p < 0.05, **p < 0.01, ***p < 0.001. MO313 cells without treatment are labeled as a control group (C).

To elucidate the impact of psychosine upon autophagy and UPS, the human oligodendrocyte cell line MO3.13 treated with psychosine was used as a cellular model to recapitulate the pathomechanism of GLD. In contrast to a previous study showing synergic effects of psychosine and starvation on the activation of autophagy in MO3.13 cells [30], our study used full medium to investigate the sole impact of psychosine on autophagic flux without the starvation-induced autophagy. MO3.13 cells were cultured in the full medium for 24 h in the presence of different concentrations of psychosine, and the expression levels of autophagic markers LC3 and p62 proteins were determined (Figure 7a). The Western blot analysis demonstrated that LC3 II protein expression was elevated progressively in the presence of increasing concentrations of psychosine compared with that of the untreated control (Figure 7b).In the presence of psychosine, the levels of LC3 II increased significantly to 1.8-, 4.8-, 7-, and 8.2-fold of those of the control at concentrations of 5, 10, 15, and 20 µM, respectively, and remarkably, to 21.2-fold of that of the control at concentration of30µM, suggesting the activation of autophagy in line with the concentration of psychosine. Consistent with the expression of LC3 II proteins, the levels of p62 also increased progressively in the presence of psychosine at concentrations of 5–15 µM, though these increases were not significant statistically, and accumulated significantly at concentrations of 20µM and 30 µM (Figure 7c), suggesting an impairment of autophagy upon degradation of autophagic substrates at high doses. This observation validates a dose-dependent cytotoxicity of psychosine upon the activation and function of autophagy.

Further, the cytotoxicity of psychosine upon cell viability was measured in MO3.13 cells treated with different concentrations of psychosine for 24 h (Figure 7d). At 5 and 10µM, psychosine did not cause cell death after the incubation for 24 h, while psychosine at 15 µM for 24 h caused 85% cell survival, albeit not significantly, and psychosine at 20 and 30 µM caused a significant decrease of cell viability to 74.2% and 56.7% cell survival at 24 h, respectively. Thus, psychosine at high concentrations (30 µM) was lethal to oligodendrocytes.

Finally, the time-dependent impact of psychosine at 20 µM for 72 h upon autophagy of oligodendrocytes was determined (Figure 7e). Western blot analysis demonstrated that LC3-II protein expression was increased significantly and progressively from 24 h to 72 h with exposure to psychosine (Figure 7f). In line with the increase of LC3-II protein expression, levels of p62 protein were also increased significantly and gradually; levels were indistinguishable between 48 h and 72 h (Figure 7g). Of note, psychosine at 20 µM caused a prominent and significant decrease of cell viability to 54.3% survival under the incubation for 72 h, indicating time-dependent cytotoxicity. (Figure 7h). Collectively, our results indicate a dose- and time-dependent cytotoxicity of psychosine upon the capacity of autophagy and cell viability of oligodendrocytes.

### 3.6. Autophagy Inhibition Exacerbates the Accumulation of p62 Aggregates

**Figure 8:**
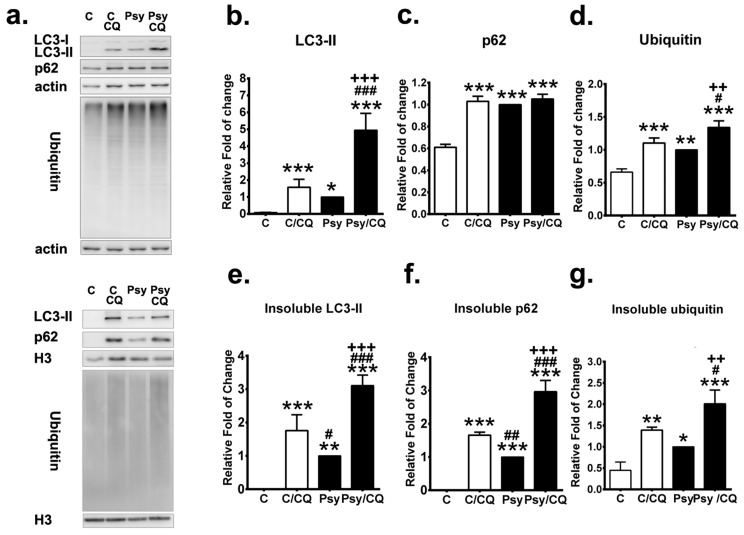
Autophagy inhibition increases accumulation of insoluble p62 and ubiquitin. (**a**) Western blot analysis of the total LC3-II, p62, and ubiquitin in MO3.13 cells treated without (control: C)/with psychosine (Psy) for 72 h concomitant with/without chloroquine (CQ) for the last 24 h. Soluble and insoluble fractions were analyzed. β-actin (actin) and histon-H3 (H3) were used for the loading control in soluble and insoluble fractions, respectively. (**b**–**d**) Quantification of soluble LC3-II (**b**), p62 (**c**), and ubiquitin (**d**). (**e**–**g**) Quantification of insoluble LC3-II (**e**), p62 (**f**), and ubiquitin (**g**). Values are expressed as mean ± S.D. (*n* = 3). Statistical significance * *p* < 0.05, **p < 0.01, *** *p* < 0.001 in comparison to control (C). C/CQ vs. Psy, or Psy/CQ, ^#^ *p* < 0.05, ^##^ *p*< 0.01,^###^*p* < 0.001. Psy vs. Psy/CQ, ^++^*p*< 0.01, ^+++^*p*< 0.001.

To elucidate further the role of impaired autophagy in the pathogenesis of GLD, autophagic flux and UPS were measured in the presence and absence of an autophagy inhibitor chloroquine in MO3.13 cells incubated with 20µM psychosine for 72 h (Figure 8a). Protein expression and solubility of LC3-II, p62, and ubiquitin were analyzed to measure the autophagy flux and UPS. As expected, chloroquine significantly increased LC3-II turnover and levels of LC3-II in both MO3.13 cells treated with and without psychosine (Figure 8b), where the increase of LC3-II expression and turnover was prominent in psychosine-treated cells with concomitant incubation of chloroquine (Figure 8b). Of note, inhibition of autophagy induced accumulation of insoluble LC3-II in MO3.13 control cells and further exacerbated the level of insoluble LC3-IIin psychosine-treated cells (Figure 8e). The level of p62 protein was also increased significantly after the treatment of chloroquine in control cells, while chloroquine did not further increase the already elevated levels of p62 protein expression in psychosine-treated cells (Figure 8c). However, inhibition of the autophagic flux by chloroquine significantly increased the insoluble p62 protein accumulation, which was more remarkable in MO3.13 cells treated with psychosine, while the level of insoluble p62 protein was nearly undetectable in untreated control cells (Figure 8f).

The level of ubiquitin was a useful parameter for measuring of the UPS process and was determined in cells treated with and without chloroquine in the presence and absence of psychosine. The significant increase of ubiquitin expression induced by psychosine was further augmented significantly after the addition of chloroquine, which also increased the expression of ubiquitin significantly in control cells (Figure 8d). Intriguingly, levels of insoluble ubiquitin were increased significantly in cells treated with psychosine, indicating a dysfunction of the UPS process, and were further accumulated significantly after the concomitant addition of chloroquine (Figure 8g). Taken together, these findings indicate that inhibition of autophagic flux further exacerbates the accumulation of LC3-II, p62, and ubiquitin in insoluble protein fractions mediated by psychosine cytotoxicity.

**Figure 9:**
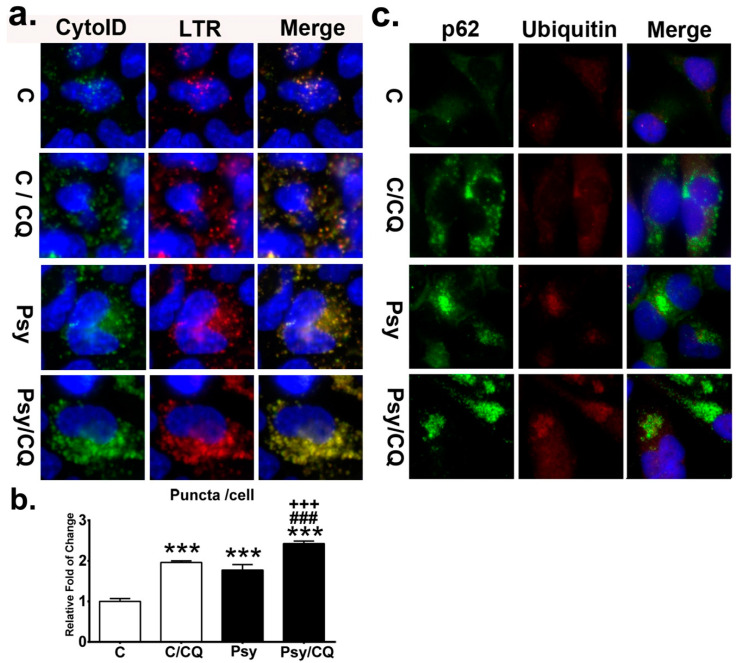
Accumulation of autolysosomes and p62 aggregates by impaired autophagic flux. (**a**) Autolysosome was determined by the colocalization of the Cyto-ID fluorescence and LysoTracker Red (LTR) in MO3.13 cells treated with/without psychosine (Psy) concomitant with/without chloroquine (CQ) and counter-stained with Hoechst. (**b**) Quantification of autolysosome puncta in cells. (**c**) Cytoplasmic deposition of p62 and ubiquitin by chloroquine. Cells were double labeled with anti-p62 and anti-ubiquitin and counterstained with DAPI. Values are expressed as mean ± S.D. (*n* = 3). Statistical significance *** *p*< 0.001 in comparison to control (C). C/CQ vs. Psy, or Psy/CQ, ^###^*p* < 0.001. Psy vs. Psy/CQ, ^+++^*p* < 0.001.

During the process of autophagy, LC3-II and p62 are incorporated into the autophagosomal membrane, which fuses with lysosomes to form autolysosomes, degrading the misfolded and/or damaged proteins [34]. Therefore, an increase in LC3-IIexpression alone does not provide a complete picture of autophagy in the cells, while observations and measurement of the concurrent formation of the autolysosome depict the autophagic flux [46]. To quantify the autophagy induced by psychosine in cells, the formation of autolysosomes was determined by staining MO3.13 cells with both Cyto-ID and LysoTracker Red for autophagosomes and lysosomes, respectively. It has to be noted that the use of fluorescent probes Cyto-ID and LysoTracker Red have been broadly applied to visualize the autophagy dynamics in live cells in many studies [47,48]. Recent studies further indicated that Cyto-ID labels autophagic compartments with minimal and negligible staining of lysosomes [47], while the LysoTracker Red labels the lysosomes specifically [49]. There is compelling evidence validating the identification of autophagosomes by CytoID, lysosomes by LysoTracker Red, and autolysosomes by colocalization of CytoID and LysoTracker Red [48,50,51]. The process of autophagic flux was thus evaluated by direct imaging and quantification of autolysosomes in cells (Figure 9a,b). The puncta of autolysosomes in cells were significantly increased from 1.8-fold to 2.4-fold compared to that of the untreated control after addition of chloroquine in psychosine-treated cells, and a 2-fold increase compared to that of the control cells treated with chloroquine (Figure 9b). Images of autolysosomes in cells showed numerous enlarged puncta accumulated in the cytoplasm after the addition of chloroquine, especially in those cells with concomitant incubation of psychosine (Figure 9a).

To evaluate whether impairment of autophagy leads to aggregation of p62 and ubiquitin as demonstrated in the twitcher brain, immunefluorescent staining for both p62 and ubiquitin was performed in MO3.13 cells with and without treatment of chloroquine in the presence and absence of psychosine (Figure 9c). It was shown that psychosine induced cytoplasmic aggregates with p62, which was more prominent with concomitant treatment with chloroquine. The control cells showed a few small puncta of p62, which was evident after the addition of chloroquine, in accordance with the inhibition of autophagy. Furthermore, small puncta of ubiquitin-positive aggregates were observed in psychosine treated MO3.13 cells and were increased moderately after the addition of chloroquine. Whereas small ubiquitin-positive puncta were scarcely observed in control cells after treatment with chloroquine. 

### 3.7. Inhibition of Both Proteasome and Autophagy Augments Aggregation with Ubiquitin and p62

**Figure 10:**
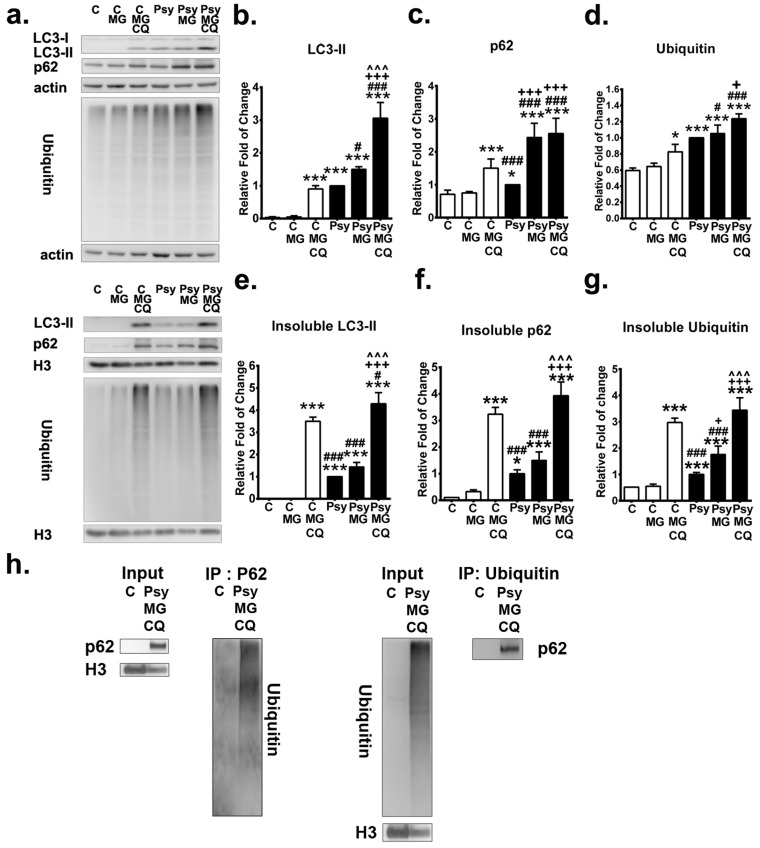
Inhibition of autophagy and proteasome exacerbates accumulation of p62 and ubiquitin. (**a**) Western blot analysis of the total LC3-II, p62, and ubiquitin in MO3.13 cells treated without (control: C)/with psychosine (Psy) for 72 h concomitant with/without MG132 (MG) with/without chloroquine (CQ) for the last 24 h. Soluble and insoluble fractions were analyzed. β-actin (actin) and histon-H3 (H3) were used for the loading control in soluble and insoluble fractions, respectively. (**b**–**d**) Quantification of soluble LC3-II (**b**), p62 (**c**)and ubiquitin (**d**). (**e**–**g**) Quantification of insoluble LC3-II (**e**), p62 (**f**) and ubiquitin (**g**). (h) Interaction between insoluble p62 and ubiquitin was verified by immunoprecipitation analysis (**h**).Values were expressed as mean ± S.D. (*n* = 3). Statistical significance *p< 0.05, ***p < 0.001 in comparison to control (C) or C/MG. C/MG/CQ vs. Psy, or Psy/MG, or Psy/MG/CQ, ^#^*p*< 0.05, ^###^*p* < 0.001. Psy vs. Psy/MG, or Psy/MG/CQ, ^+^*p* < 0.05, ^++^*p* < 0.01, ^+++^*p*< 0.001. Psy/MG vs. Psy/MG/CQ, ^^^^^*p* < 0.001. No statistical significance between C and C/MG.

The impact of UPS dysfunction upon induction of p62- and ubiquitin-positive aggregates as observed in the twitcher brain was further investigated in the MO3.13 cellular model, which was exposed to the proteasome inhibitor MG132 at a subtoxic concentration (Figure 10a). It has been shown that MG132potently inhibits the chymotrypsin-like activity of the proteasome, leading to exacerbation of the burden of misfolded and damaged proteins and concomitant cellular stress [52]. At a subtoxic concentration, MG132 did not increase the levels of LC3-II and p62 protein expression in the soluble fraction of control cells but increased the level of insoluble p62 protein (Figure 10b, 10c, 10e, 10f). While in the psychosine-treated cells, MG132 significantly increased the expression of LC3-II and p62 proteins, which were further accumulated significantly in the insoluble phase (Figure 10b,c,e,f). Consistently, the level of ubiquitin in control cells, in either the soluble or insoluble phase, was not altered by a subtoxic concentration of MG132 (Figure 10d,g). Of note, MG132 increased the accumulation of insoluble ubiquitin significantly from an initial 2-fold of control to 4-fold of control in psychosine-treated cells (Figure 10g).

Moreover, the effect of inhibition of both autophagy and proteasomes by concomitant treatment of chloroquine and MG132 was determined (Figure 10a). Levels of LC3-II, p62, and ubiquitin proteins were significantly increased in both the soluble and insoluble phase in control cells under the simultaneous inhibition of autophagy and proteasomes (Figure 10b,g). In psychosine-treated cells, the concomitant addition of both chloroquine and MG132 induced a significant increase of LC3-II in both the soluble and insoluble phase and a robust accumulation of insoluble p62 and insoluble ubiquitin proteins (Figure 10b,g). Further, the binding of insoluble p62 and ubiquitin was confirmed by the immunoprecipitation analysis (Figure 10h).

**Figure 11:**
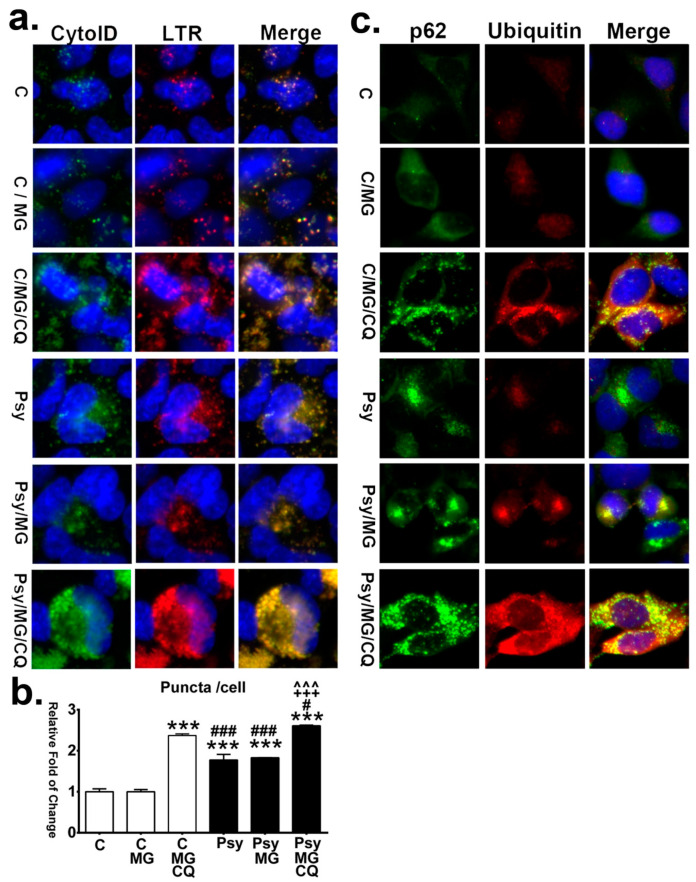
Impaired autophagic flux and proteasomes enhance aggressive accumulation of autolysosomes and aggregation of p62 and ubiquitin. (**a**) Autolysosome was determined by the colocalization of the Cyto-ID fluorescence and LysoTracker Red (LTR) in MO3.13 cells treated with/without psychosine (Psy) concomitant with/without MG132 (MG) with/without chloroquine (CQ), and counter-stained with Hoechst. (**b**) Quantification of autolysosome puncta in cells. (**c**) Increased p62- and ubiquitin-aggregation. Cells were double labeled with anti-p62 and anti-ubiquitin, and counterstained with DAPI. Values are expressed as mean ± S.D. (*n* = 3). Statistical significance ***p< 0.001 in comparison to control (C) or C/MG. C/MG/CQ vs. Psy, or Psy/MG, or Psy/MG/CQ, ^#^*p* < 0.05, ^###^*p* < 0.001. Psy vs. Psy/MG, or Psy/MG/CQ, ^+++^*p* < 0.001. Psy/MG vs. Psy/MG/CQ,^^^^^*p* < 0.001. No statistical significance between C and C/MG.

Direct observation and quantification of autolysosomes in cells showed that MG132 alone did not further increase the accumulation of autolysosomes in psychosine-treated cells, while concomitant treatment with chloroquine and MG132 increased the accumulation of autolysosomes significantly in both control and psychosine-treated cells (Figure 11a,b). Furthermore, with immunofluorescent labeling of p62 and ubiquitin, it was demonstrated that the signal of ubiquitin-positive puncta became intense after the addition of MG132 in both control and psychosine-treated cells (Figure 11c). Of note, large pleomorphic aggregates containing ubiquitin and p62 proteins were observed in psychosine-treated cells with concomitant inhibition of autophagy and UPS, recapitulating the pathological hallmarks observed in the twitcher brain in this study. 

### 3.8. Inhibition of Both Proteasome and Autophagy Leads to Accumulation of ROS, Reduction of Mitochondrial Respiration, and Decreased Viability

**Figure 12:**
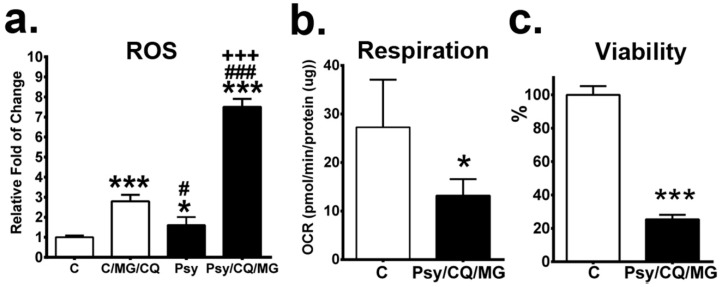
Inhibition of both proteasome and autophagy promotes cell death. MO3.13 cells treated without/with psychosine (Psy) for 72 hs concomitant with MG132 (MG) and chloroquine (CQ) in the last 24 h. (**a**–**c**) Levels of ROS (**a**), basal respiration (**b**), and cell viability (**c**) were quantified. Values areexpressed as mean ± S.D. (ROS, *n* = 3; Respiration, *n* = 5; Viability, *n* = 6). Statistical significance *p < 0.05, ***p < 0.001 in comparison to control (**C**). C/MG/CQ vs. Psy, or Psy/MG/CQ, # *p* < 0.05, ### *p* < 0.001. Psy vs. Psy/MG/CQ, +++ *p* < 0.001.

The consequence of the inhibition of proteasomes and autophagy was further explored by identifying the levels of reactive oxygen species (ROS), a function of mitochondrial respiration and cell viability. Our results showed that inhibition of autophagy and UPS induced a prominent increase of ROS levels in psychosine-treated MO3.13 cells in comparison to that of control cells (Figure 12a). Furthermore, the assay of mitochondrial respiration by direct analysis of oxygen consumption rate demonstrated a significant reduction of respiration in MO3.13 cells treated with psychosine concomitant with inhibition of autophagy and UPS (Figure 12b). Accordingly, the cell viability of psychosine treated MO3.13 cells with inhibition of autophagy and UPS was remarkably decreased to 25.4% of that of control cells (Figure 12c).

## 4. Discussion

Cytotoxicity of psychosine has been implicated in the pathological hallmarks of GLD, inducing demyelination, astrocytosis, and formation of multinuclear globoid-like cells in the central nervous system (CNS) [25,27,53,54,55]. Progressive accumulation of psychosine upregulates the production of inflammatory cytokines and generation of lysophosphatidylcholine and arachidonic acid resulting in apoptosis and cell death in oligodendrocytes [28,53,54,56]. However, the underlying mechanism of impact of psychosine on oligodendrocytes contributing to demyelination of GLD still remains largely elusive. In this study, we provide the first evidence, in vivo and in vitro, that psychosine induces impairment of UPS and autophagy, resulting in cytoplasmic accumulation of insoluble ubiquitin- and p62-aggregates, giving rise to an elevation of ROS and cell death. The spatial-temporal distribution of ubiquitin- and p62-aggregates correlates with progressive demyelination in the nervous system and provides connection to the neurological symptomatology.

Though misfolded and damaged proteins mediated by psychosine have been implicated in the neuropathy of GLD, the impact of imbalanced proteostasis upon oligodendrocytes and white matter has not been validated. Previous studies demonstrated that psychosine interacts with α-synuclein and promotes aggregation of fibrillized α-synuclein in neurons in the brains of both human and twitcher mice [23]. A recent study further demonstrated a deposition of p62 aggregates in neurons of the twitcher brain at an early symptomatic stage, albeit the locations of neuronal aggregates in regions of the twitcher brain were not indicated and presence of p62 aggregates in oligodendrocytes was not documented [29]. These findings suggest that misfolded protein aggregates are involved in the pathogenesis of GLD. In this study, we first demonstrated that the ubiquitin- and p62-aggregates, which colocalize with oligodendrocytes, accumulate ubiquitously and constitutively in white matter and parallel the progressive demyelination of disease progression. Additionally, neurons with ubiquitin- and p62-aggregates were moderate and localized in the cerebellar granular layer, brain stem, and spinal cord. Cytosolic aggregates containing ubiquitinated proteins and p62 proteins have been linked to dysfunction of UPS and autophagy, underscoring the pathogenesis of many neurodegenerative diseases and several demyelination disorders [35,36,37,57]. Soluble extraneous or misfolded proteins are ubiquitinated and directed to UPS for degradation, while decreased efficacy or overwhelmed proteasome systems lead to soluble ubiquitinated proteins and insoluble aggregates, which become cellular inclusions, as a hallmark of neurodegenerative proteinopathies. Damaged protein, organelles, and insoluble aggregates are engulfed in autophagosomes, which are decorated with LC3 and p62 for maturation and subsequent lysosomal degradation, whereas dysfunction of autophagy results in the formation of insoluble cytosolic aggregates associated with p62 accumulation, contributing to the pathogenesis of neurodegenerative diseases and several lysosomal storage diseases [38,58]. Of note, p62 also serves as an adaptor protein, linking UPS and autophagy by directly binding ubiquitinated proteins for degradation in autophagy [59,60]. The cytosolic accumulation of aggregates containing both ubiquitin and p62 proteins has been widely associated with compromised UPS and autophagy in many neurodegenerative diseases. Along these lines, our findings indicate lysosomal GALC deficiency contributing to the dysfunction of UPS and autophagy, which underlies the vulnerability to demyelination and neurodegeneration in GLD.

To address the role of autophagy implicated in the pathogenesis of demyelination in GLD, several human and murine cell lines with exogenous supplements of psychosine have been used as a disease-cell model [9,21,22,23,24,25,26,27,28,29,30,31]. In a study by Ribbens et al., murine oligodendrocyte cell lines derived from the twitcher brain showed elevated levels of LC3-II colocalized with lysosomes under exogenous treatment with 15µM psychosine for 24 h, suggesting induction of autophagy by psychosine [24]. Another study by Del Grosso et al. demonstrated an evident autophagy activation in the human oligodendrocyte cell line MO3.13 under the synergic impact of 10 µM psychosine and serum starvation for 24 h [30]. Furthermore, Del Grosso et al. showed that levels of p62 and LC3-II were elevated in twitcher fibroblasts with exposure to 100 µM, psychosine, albeit higher levels of p62 protein were also noted in psychosine-untreated twitcher fibroblasts and levels of p62 aggregates were indistinguishable between psychosine-treated and untreated twitcher cells [29]. Those previous studies all demonstrated an activation of autophagy under short-term exposure to psychosine at different concentrations in different disease-cell models. Intriguingly, in the present study using the human oligodendrocyte cell line MO3.13, we are the first to document a disease-cell model recapitulating the pathogenesis of GLD. Our results indicate that psychosine induces impairment of autophagy and UPS with a dose-and time-dependent cytotoxicity, which explains the activation of autophagy under short-term exposure of psychosine in previous studies [24,29,30]. Elevated concentrations of psychosine elicit an increase of autophagy, progressive and prolonged treatment of psychosine (20 µM for 72 h) disrupts the function of both autophagy and UPS, characterized with the accumulation of LC3-II, p62, and ubiquitin proteins in the insoluble fraction. Of note, inhibition of proteasomes and autophagy further exacerbates the accumulation of insoluble proteins p62 and ubiquitin, mediated by psychosine, and augments cytoplasmic deposition of p62- and ubiquitin-aggregates, which recapitulate the pathological findings observed in the twitcher brain in this report. Those findings implicate a time-dependent accumulation of p62- and ubiquitin-aggregates by psychosine cytotoxicity, supporting the chronological changes of pathogenesis observed in the brain of twitcher mice.

In the present study, our findings provide evidence for a link between psychosine and dysfunction in autophagy and UPS, which underlies vulnerability to progressive demyelination in GLD. Protein homeostasisis regulated by the crosstalk between UPS and autophagy, while dysfunction of autophagy and/or UPS leads to cytoplasmic deposition of p62- and ubiquitin-aggregates, underlying the molecular mechanism for neurodegenerative diseases. It is argued that the aggregates of p62 and ubiquitin in the twitcher brain are the sequential results from impairment of autophagy or UPS alone or combination. In the disease-cell model, psychosine induced a decrease of proteasome activity and accumulation of insoluble ubiquitin and cytoplasmic aggregates, indicating the impairment of UPS is caused by the psychosine toxicity. Several reports have documented mechanisms on how aggregated proteins interact with proteasomes and impair the function of UPS. It has been shown that heterogeneous aggregates of prion proteins impair UPS though reducing proteasomal gating for substrate entry into protealytic sites [61]. A recent study further validates that aggregate proteins generated from Alzheimer’s disease, Parkinson’s, and Huntington’s disease form three-dimensional oligomers, which inhibit proteasome gates opening, thus impairing ubiquitin-dependent and ubiquitin-independent proteasome function [62]. These findings provide a partial explanation to psychosine-induced proteasome impairment, given that psychosine accelerates the formation of α-synuclein oligomers [23].

In regards to maintaining protein homeostasis, UPS impairment upregulates autophagy to compensate for the dysfunction of the proteasome [63,64,65,66]. Indeed, we observed an increase of LC3-II both in vivo and in vitro, suggesting an increment of autophagic flux, while the concomitant accumulation of p62 as an indicative marker of dysfunction of autophagy was also demonstrated in our study. The increased expression of p62can compromise the protein homeostasis of misfolded proteins, leading to the formation of ubiquitinated protein aggregates [67,68]. A previous study indicated that increased expression of p62 in expanded polyglutamine-expressing cells with inhibition of proteasome enhanced the formation of aggresomes and ubiquitinated polyglutamine inclusions [68]. Moreover, over expression of p62 accelerates the accumulation of insoluble p62 and poly-ubiquitinated proteins by hindering the function of UPS and autophagy in the degradation of misfolded proteins [67]. In the present study, the accumulation of insoluble p62 and cytoplasmic deposition of p62 aggregates in the twitcher brain and disease-cell model implicated the impairment of the autophagy pathway. Several molecular mechanisms have been linked to dysfunction of autophagy. The disruption of autophagic flux observed in the twitcher brain can be caused by an overload of autophagic substrates above the capacity of degradation or multiple lysosomal dysfunctions mediated by psychosine [69,70]. Similar to the pathogenesis of Niemann–Pick Type C, we observed an accumulation of autophagosomes, autolysosomes, and p62 in our disease-cell model, suggesting the reduction of lysosomal activity and subsequent impairment of autolysosomal cargo degradation [39]. Previous studies have indicated that psychosine disrupts lysosomal function by causing abnormalities in lipid homeostasis, endolysosomal transport, and cathepsin activity [69]. Additionally, lysosomal membrane permeability caused by the primary lysosomal deficit, which disrupts the efficacy of lysosomal degradation, promotes insoluble p62 aggregate formation into cytosolic inclusions [58]. Nonetheless, excess accumulation of p62 by impaired autophagy can hinder the trafficking of ubiquitinated proteins to the proteasome proteases for degradation, resulting in reduced degradation of UPS substrates and accumulation of aggregation-prone proteins [71]. Collectively, reciprocal regulation of autophagy and UPS is crucial for the maintenance of protein homeostasis, while an imbalance between both proteolytic pathways caused by either system alone or both systems together could result in the same pathological consequence. Accordingly, misfolded protein accumulation and aggregation cause over-production of reactive oxygen species, which contributes to cell death. 

## 5. Conclusions 

Our study, for the first time, unveils the dysfunction of UPS and autophagy contributing to the pathogenesis of demyelination and neuropathy in GLD. Here, we describe the increase of insoluble p62 and ubiquitin proteins concomitant with spatiotemporal accumulation of colocalized ubiquitin- and p62-aggregatesin alignment with a decrement of myelin protein, which underlies vulnerability to progressive demyelination in the murine model of GLD. Impairment of proteasomes and autophagy results in the accumulation of ROS, reduction of mitochondrial respiration, and eventually decreased viability. These findings provide new insights in the understanding of the molecular mechanism for the pathogenesis of GLD, and provide possible therapeutic avenues via targeting brain regions with specific pathomechanisms, modulation of proteostasis, as well as providing a sufficient antioxidant system. 

## Figures and Tables

**Table 1:** List of primary antibodies for immunohistochemistry (IHC), immunocytochemistry (ICC), and Western blot (WB) analyses.

Antibodies	Host	Supplier	IHC Dilution	ICC Dilution	WB Dilution
Anti-LC3B	Rabbit	Sigma-Aldrich (St. Louis, MO, USA)			1:1000
Anti-P62	Mouse	Abcam (Cambridge, UK)	1:300		1:2000
Anti-P62	Rabbit	Proteintech (Chicago, IL, USA)	1:300	1:300	
Anti-Ubiquitin	Mouse	Abcam (Cambridge, UK)	1:300	1:300	
Anti-Ubiquitin	Rabbit	Cell Signaling (Danvers, MA, USA)	1:200		1:1000
Anti-Nrf2	Rabbit	Abcam (Cambridge, UK)			1:1000
Anti-Keap1	Rabbit	Proteintech (Chicago, IL, USA)			1:1000
Anti-NQO1	Rabbit	Genetex (Irvine, CA)			1:1000
Anti-Histone H3	Mouse	Cell Signaling (Danvers, MA, USA)			1:1000
Anti-β-Actin	Mouse	Sigma-Aldrich (St. Louis, MO, USA)			1:5000
Anti-NeuN	Mouse	Millipore (Burlington, MA, USA)	1:100		
Anti-Calbindin	Mouse	Sigma-Aldrich (St. Louis, MO, USA)	1:100		
Anti-GFAP	Rabbit	Dako (Santa Clara, CA)	1:200		
Anti-GFAP	Mouse	Invitrogen (Eugene, OR, USA)			1:2000
Anti-MBP	Mouse	Abcam (Cambridge, UK)	1:100		
Anti-MBP	Mouse	Millipore (Burlington, MA, USA)			1:1000
Anti-PLP	Rabbit	Abcam (Cambridge, UK)	1:100		
Anti-PLP	Mouse	Abcam (Cambridge, UK)	1:100		
Anti-Iba1	Rabbit	Biocare (Pacheco, CA, USA)	1:100		
Anti-LAMP1	Rabbit	Genetex (Irvine, CA)	1:100		

## Abbreviations

ATG	autophagy-related proteins;
GFAP	glial fibrillary acidic protein;
GLD	globoid cell leukodystrophy;
Iba-1	ionized calcium-binding adapter molecule 1;
LC3	microtubule-associated protein 1 light chain;
LC3-II	LC3-phosphatidylethanolamine conjugate;
LSD	lysosomal storage disease;
LAMP1	lysosomal-associated membrane protein 1;
MBP	myelin basic protein;
NeuN	neuronal nuclei;
PLP	proteolipd protein;
Twi	twitcher mice;
UPS	ubiquitin proteasome system;
WT	wild-type mice.
